# Bilateral Posterior Subthalamic Area Deep Brain Stimulation for Essential Tremor: A Case Series

**DOI:** 10.3389/fnhum.2020.00016

**Published:** 2020-02-05

**Authors:** Xiaoyu Sun, Luke Huang, Yixin Pan, Chencheng Zhang, Tao Wang, Hongxia Li, Bomin Sun, Jianqing Ding, Yiwen Wu, Dianyou Li

**Affiliations:** ^1^Department of Functional Neurosurgery, Ruijin Hospital, School of Medicine, Shanghai Jiao Tong University, Shanghai, China; ^2^Department of Neurology, Ruijin Hospital, School of Medicine, Shanghai Jiao Tong University, Shanghai, China

**Keywords:** essential tremor, deep brain stimulation, posterior subthalamic area, ventral intermediate nucleus of the thalamus, head tremor

## Abstract

**Background:**

Deep brain stimulation (DBS) of the posterior subthalamic area (PSA) provides a potentially effective treatment for medication-refractory essential tremor (ET).

**Objective:**

To study the clinical benefits and adverse-event profile of bilateral PSA-DBS for refractory ET.

**Methods:**

Seven patients with refractory ET underwent bilateral PSA-DBS surgery under general anesthesia between September 2017 and May 2018. Clinical outcome assessments, using the Essential Tremor Rating Scale, were performed at 1-, 6-, and 12-month follow-up, except for the last assessment of one patient who was followed up to 9 months. Analysis was focused on changes in patients’ motor symptoms and quality of life following surgery as well as documenting the adverse-event profile associated with the surgical PSA-DBS treatment.

**Results:**

After surgery, patients’ motor symptoms, including upper limb tremor and head tremor, were improved by 84.2% and their quality of life by 81.25% at 1-month follow-up. The clinical benefits to patients were maintained at 6-month and last follow-up. Adverse side effects included dysarthria (*n* = 4), balance disorder (*n* = 2), and paresthesia of the right limb (*n* = 1). No habituation effects were observed throughout the follow-up.

**Conclusion:**

Bilateral PSA-DBS seems to offer an effective and safe alternative treatment for medically intractable ET, warranting further research into its clinical utility, adverse-event profile, and comparative effectiveness.

## Introduction

Essential tremor (ET) is a relatively common movement disorder associated with marked physical and psychosocial disabilities (Louis and Ferreira, 2010). Deep brain stimulation (DBS) has emerged as a safe and effective treatment for medically refractory ET (Anderson and Kartha, 2013). The ventral intermediate nucleus of the thalamus (VIM) has been mainly used as the target

1in DBS treatment for ET, and its stimulation is particularly effective in reducing hand tremor (Flora et al., 2010). However, the effects of DBS on midline symptoms, such as head tremor, have been inconsistent across studies and are less predictable. It has been reported that bilateral VIM-DBS reduces head tremor and that bilateral stimulation is more effective than unilateral DBS (Obwegeser et al., 2000). However, bilateral VIM-DBS is associated with a higher risk of adverse side effects than unilateral VIM-DBS, including dysarthria, incoordination, and abnormal gait; DBS reprograming within the therapeutic window may not resolve these side effects (Mitchell et al., 2000). Moreover, 10–73% of patients who underwent VIM-DBS, particularly bilateral DBS, seems to develop observable tolerance and waning of benefits over the long-term treatment course (Benabid et al., 1996, 1998; Papavassiliou et al., 2004; Pilitsis et al., 2008; Shih et al., 2013). Given these limitations, several DBS studies have explored the utility of targets other than the VIM in treating ET.

Kitagawa et al. (2000) reported a case of ET treated with unilateral DBS of a target positioned 3 mm below the VIM. After treatment, the patient’s postural tremor was substantially improved. Another target of interest is the posterior subthalamic area (PSA), which includes the zona incerta and prelemniscal radiation. Emerging evidence from PSA-DBS studies (Herzog et al., 2007; Patric et al., 2009; Blomstedt et al., 2010, 2011; Barbe et al., 2011, 2018; Fytagoridis et al., 2012; Ulrika et al., 2012) has indicated that stimulation of this target may be effective in reducing tremor, particularly when the tremor is difficult to control with VIM-DBS. Moreover, the adverse side effects of PSA-DBS seem to be mild and transient, without enduring side effects or stimulation tolerance (Pahwa et al., 2006; Fytagoridis and Blomstedt, 2010). To date, however, only a few studies have been conducted to assess the safety and effectiveness of PSA-DBS for ET, and even fewer studies have explored the clinical effects of bilateral PSA stimulation (Xie et al., 2012; Ghilardi et al., 2018). Therefore, we retrospectively assessed the clinical outcomes of a series of patients with medically refractory ET who underwent bilateral PSA-DBS. The study results should contribute to a better understanding of the clinical benefits and adverse-event profile of bilateral PSA-DBS in patients with refractory ET.

## Materials and Methods

In this retrospective case series, seven patients were identified from past medical records and followed up to the present. Each patient was clinically assessed and videotaped at regular intervals as part of standard care after they had received bilateral PSA-DBS surgery. Six patients were followed up for 12 months and one patient was followed up for 9 months. The average follow-up duration of the patient sample was 11.6 months. At the last follow-up, participating patients gave written informed consent for this study. The study was approved by the ethics committee of Ruijin Hospital School of Medicine, Shanghai Jiao Tong University, and was performed in accordance with the Declaration of Helsinki.

### Surgical Procedure

**FIGURE 1:**
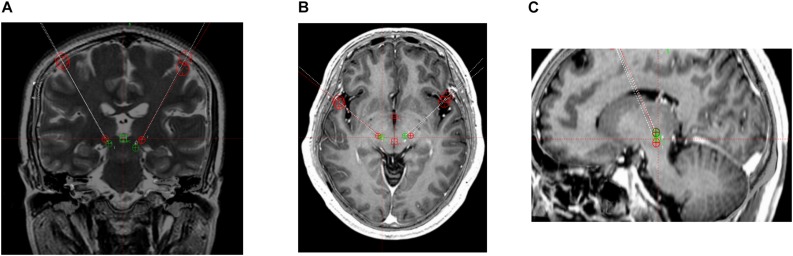
3 T magnetic resonance images. Target coordinates of the posterior subthalamic area (PSA) were 10 mm lateral, 7.7 mm anterior, and 3.4 mm superior. PSA (green) and ventral intermediate nucleus of the thalamus (red) contacts. **(A)** Coronal T2-weighted image. **(B)** Horizontal T1-weighted image. **(C)** Sagittal T1-weighted image.

**FIGURE 2:**
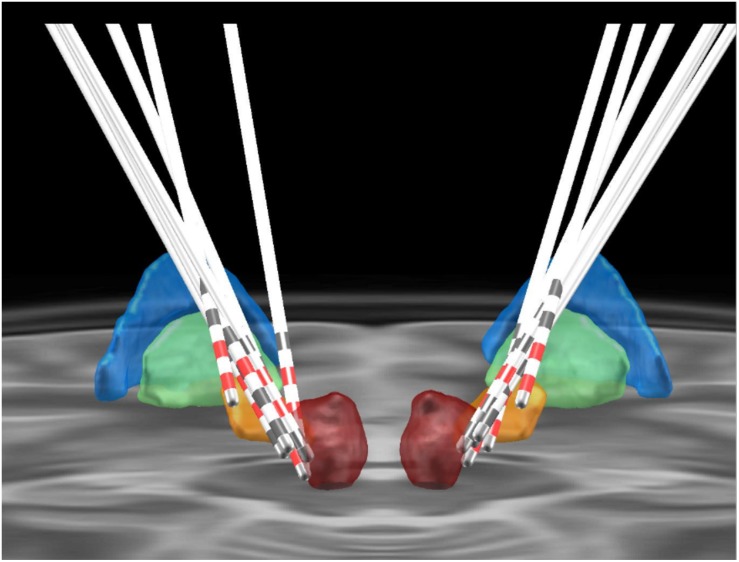
Positioning of the electrodes for posterior subthalamic area-deep brain stimulation. The colored areas represent different anatomical structures (blue, globus pallidus externus; green, globus pallidus internus; orange, subthalamic nucleus; red, red nucleus).

The surgical procedure included target localization and implantation of electrodes and pulse generators. Patients underwent 3.0-T magnetic resonance imaging (MRI) involving continuous scanning in the horizontal and coronal planes (slice thickness, 2 mm) and T2-weighted and post-gadolinium volumetric axial T1-weighted sequences. VIM targeting began with identifying the standard stereotactic coordinates relative to the posterior commissure (PC) on an anterior–posterior commissure (AC-PC)-aligned MRI; the position was 10.5–11.0 mm lateral to the wall of the third ventricle, 6.0–7.0 mm anterior, and 0 mm dorsal (Leksell Surgiplan, version 10.0, Elekta Instrument AB, Stockholm, Sweden). Subsequently, the PSA target was identified as being medial to the posterior tail of the subthalamic nucleus and lateral to the red nucleus on an AC-PC-aligned axial T2-weighted MRI at the level of the widest diameter of the red nucleus (Figures 1, 2).

Our strategy was to target the VIM and PSA if both targets could be targeted. The trajectory was planned by using the PSA as the primary target. The coronal and sagittal angles were adjusted to attain a trajectory that traversed the VIM target. The planned trajectory was identified in coronal sections, beginning at the top of a gyrus and avoiding the ventricles, caudate nucleus, and blood vessels. The PSA was targeted in cases where the planned alignment was not achievable. In four patients, both the VIM and PSA were targeted. In the other three patients, both regions were not simultaneously targeted because we were unable to align them in one trajectory (Bot et al., 2017).

A stereotactic frame (Leksell) was installed under local anesthesia. The stereotactic pedestal was placed in parallel with the front and rear joint line (AC-PC). The head-computed tomography image was transmitted to the surgical planning system to determine the PSA target coordinates. Next, electrodes (3387, Medtronic or L102, PINS, Beijing Pins Medical Equipment Co., Ltd., Beijing, China) were implanted under general anesthesia. A pulse generator (37612 RC Medtronic or 102R PINS) was implanted under the clavicle. One week after DBS electrode implantation, the images of the patient’s head and the preoperative MRI (using a Helix sequence) were merged to determine the position of the electrodes before programing. In this study, we focused on assessing the clinical effects of PSA stimulation; only the electrodes located in the PSA were activated.

### Clinical Examination

In each patient, DBS was activated 1 week after surgery. We examined the clinical effects of stimulation after the location of each electrode was confirmed. Electrodes that displayed the best stimulation effect were selected for chronic stimulation. As outlined in the prior section, we focused on the electrodes located in the PSA.

A movement disorder specialist performed the clinical outcome assessments, using the Essential Tremor Rating Scale (ETRS) (Fahn et al., 1988), before surgery and at 1-month, 6-month, and last (9- or 12-month) follow-up. Assessments focused on motor symptoms (items 1–9 of the ETRS), quality of life (items 15–21 of the ETRS), and complications, such as dysarthria, balance disorders, and hemiplegia. Additionally, the patients were videotaped with and without stimulation (stimulation was switched off during the preceding night) by another movement disorder specialist who was blinded to the patients’ condition. This specialist also examined the videos.

### Statistical Analysis

We performed a Kolmogorov–Smirnov test and the results indicated that nearly all variables were normally distributed and therefore suitable for parametric tests. However, it is difficult if not impossible to extrapolate data from a case series of seven patients to the overall population. Therefore, we utilized (non-parametric) Wilcoxon signed rank tests to make comparisons between the ETRS scores acquired under the different conditions. *P*-values less than 0.05 (two-sided) were considered statistically significant. The analysis was performed using SPSS 23 (IBM Corp, Armonk, NY, United States). Continuous variables are described as medians and interquartile ranges for parametric and non-parametric data distributions, respectively. The results are presented as the median ± interquartile range.

## Results

### Patients

The seven patients (six male, one female; mean age: 56.1 ± 14.3 years, Table 1) had undergone bilateral PSA-DBS at the Neurosurgical Center of Shanghai Ruijin Hospital between 2017 and 2018. At the time of surgery, all patients showed bilateral upper extremity postural tremor. Three out of the seven patients also displayed head tremor. Each patient was diagnosed with ET by using the diagnostic criteria of the Movement Disorder Society (Bhatia et al., 2018), including a history of therapeutic failure of at least one first-line medication. After clinical examination, the patients underwent bilateral PSA-DBS surgery by a medical team of movement disorder specialists and functional neurosurgeons.

**TABLE 1:** Patient characteristics (*N* = 7)*.

Sex (men/women)	6/1
**Age (years)**	
Age of onset (years)	39.00 ± 9.00 (19–46)
Age of surgery (years)	59.00 ± 21.00 (29–69)
Duration (years)	20.00 ± 11.00 (7–30)
Previous VIM-DBS surgery	1 (bilateral)
Follow-up duration (months)	9 (1/7) or 12 (6/7)

VIM-DBS, ventral intermediate nucleus-deep brain stimulation. *Data values represent median ± interquartile range (min–max) unless indicated otherwise.

### ETRS Data

The patients’ ETRS scores before and after bilateral PSA-DBS surgery are presented in Tables 2, 3. At 1-month follow-up, the patients’ motor symptoms were improved, on average, by 84.2%, head tremor by 100.0%, upper limb tremor by 76.0%, and quality of life by 81.25%. These clinical benefits were significant and maintained at 6-month and last (9- or 12-month) follow-up (Table 2). No significant differences existed between the mean ETRS scores obtained at 6-month and last follow-up.

**TABLE 2:** Essential tremor rating scale scores before and after bilateral PSA-DBS (*N* = 7)*.

**Item**	**Top score**	**Baseline**	**One month**		**Improvement**	***p*-Value**	**Six months**		**Improvement**	***p*- Value**	**Last follow-up^#^**		**Improvement**	***p*-Value**
			**DBS ON**	**DBS OFF**			**DBS ON**	**DBS OFF**			**DBS ON**	**DBS OFF**		
Tremor rate (items 1–9)	80	19.00 ± 10.00	3.00 ± 4.00	14.50 ± 13.00	84.21%	0.018	2.00 ± 3.00	14.50 ± 14.50	89.47%	0.018	2.00 ± 2.00	14.50 ± 14.50	89.47%	0.018
Head tremor (item 4)	4	1.00 ± 2.25	0.00.±0.00	1.00 ± 2.00	100.00%	0.048	0.00 ± 0.00	1.00 ± 2.00	100.00%	0.048	0.00 ± 0.00	1.00 ± 2.00	100.00%	0.048
Upper tremor (items 5, 6)	24	12.50 ± 3.00	3.00 ± 2.00	11.00 ± 3.50	76.00%	0.018	2.00 ± 2.00	11.00 ± 3.50	84.00%	0.018	1.00 ± 1.50	11.00 ± 4.50	92.00%	0.018
Rest	8	4.50 ± 5.00	0.00 ± 0.00	3.50 ± 4.50	100.00%	0.031	0.00 ± 0.00	3.50 ± 4.50	100.00%	0.031	0.00 ± 0.00	3.50 ± 4.50	100.00%	0.031
Posture	8	5.00 ± 1.50	1.00 ± 1.50	5.00 ± 1.50	80.00%	0.017	1.00 ± 1.50	4.50 ± 1.50	80.00%	0.018	0.00 ± 1.50	4.50 ± 1.50	100.00%	0.017
Action	8	5.50 ± 1.50	1.00 ± 2.50	4.50 ± 1.50	81.82%	0.018	1.00 ± 2.00	4.50 ± 1.50	81.82%	0.018	1.00 ± 2.00	4.50 ± 1.50	81.82%	0.018
Quality of life (items 15–21)	28	16.00 ± 9.00	3.00 ± 5.00	15.00 ± 9.00	81.25%	0.017	3.00 ± 4.00	14.00 ± 8.00	81.25%	0.017	2.00 ± 4.00	14.00 ± 9.00	87.50%	0.017
Speech (item 15)	4	0.00 ± 1.00.	0.00 ± 1.00	0.00 ± 1.00	–	0.317	0.00 ± 0.00	1.00 ± 1.00	–	0.083	0.00 ± 0.00	1.00 ± 1.00	–	0.083

DBS, deep brain stimulation. Improvement refer to symptom change from baseline to follow-up with stimulation on. * Data values represent median ± interquartile range unless indicated otherwise. ^#^Six patients were followed up to 12 months; 1 patient was followed up to 9 months.

**TABLE 3:** Essential tremor rating scale raw scores of each patient (DBS ON).

**Patient**	**Head tremor (item 4)**				**Upper tremor (items 5, 6)**			
	**Baseline**	**One month**	**Six months**	**Last follow-up**	**Baseline**	**One month**	**Six months**	**Last follow-up**
1	2	0	0	0	14.5	1	1	0
2	0	0	0	0	15	3	3	2
3	2	0	0	0	11	5	3	3
4	0	0	0	0	12	5.5	4	4
5	0	0	0	0	12	3	3	3
6	3	0	0	0	18.5	0	1	0
7	2	0	0	0	12.5	1	1	1

### Electrode Position and Parameters

One week after surgery, the DBS parameters were set to the following values: pulse voltage = 1.5–2 V, frequency = 145 Hz, and duration = 60 μs, using monopolar stimulation. Subsequently, the stimulation parameters were adjusted according to the patients’ symptoms and extent of adverse side effects in an effort to achieve the clinically best outcomes (Table 4). Ultimately, the stimulation parameters were set to 1.75–3.35 V, 115–160 Hz, and 50–80 μs (the Pins device pulse began at 30 μs).

**TABLE 4:** Electrode position and final stimulation parameters.

**Patient**	**Contact**	**Target**	**Voltage (V)**	**Frequency (Hz)**	**Pulse (μs)**
1	C + 4−	L-PSA	1.75	125	60
	C + 0−	R-PSA	2.45	115	60
2	C + 8−9−	L-PSA	2.5	160	60
	C + 0−1−	R-PSA	2.5	160	60
3	C + 8−9−	L-PSA	3.0	145	60
	C + 1−	R-PSA	2.35	145	60
4	C + 6−	L-PSA	2.15	135	50
	C + 2−	R-PSA	2.0	135	50
5	C + 6−5−	L-PSA	3.35	135	60
	C + 1−0−	R-PSA	2.15	135	60
6	C + 6−5−	L-PSA	2.65	145	80
	C + 1−	R-PSA	2.35	145	60
7	C + 5−6−	L-PSA	2.35	145	60
	C + 1−2−	R-PSA	2.65	145	80

*PSA, posterior subthalamic area; R, right; L, left.*

### Adverse Side Effects

**FIGURE 3:**
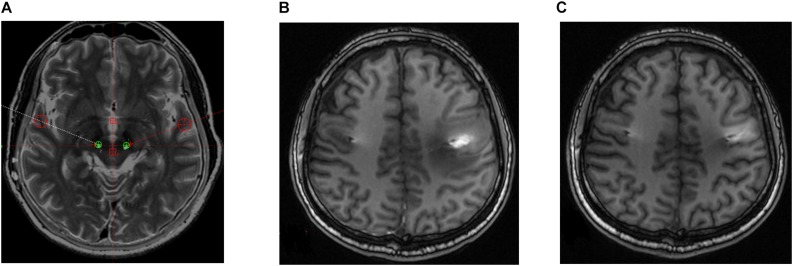
Postoperative brain magnetic resonance (MR) images in a patient who developed right paralysis due to encephaledema. **(A)** Position of electrodes. **(B)** MR image taken 1 week postoperatively. **(C)** MR image taken 8 weeks postoperatively.

Four out of the seven patients displayed mild dysarthria associated with the stimulation. Two patients in the sample developed a mild balance disorder. Both adverse side effects were resolved during the off-state condition at 6-month follow-up and did not reappear thereafter. One patient in the sample developed postoperative paralysis in the right arm due to edema in the trajectory of the left lead; this adverse event was similarly resolved at 6-month follow-up (Figure 3).

## Discussion

In this study, we explored the clinical benefits and adverse event-profile of bilateral PSA-DBS in several patients with medication-refractory ET. The results showed that bilateral PSA-DBS was associated with a significant improvement in patients’ motor symptoms, particularly upper limb tremor and quality of life. These clinical benefits were evident at 1-month follow-up and maintained at 6-month and final (9- or 12-month) follow-up. At final follow-up, patients’ motor symptoms were reduced by almost 90% compared with the severity of their symptoms before DBS treatment. Interestingly, bilateral PSA-DBS was also associated with a marked improvement in head tremor. The latter finding compares favorably with the 30–57% improvement in head tremor seen after unilateral VIM-DBS and with the 51–86% improvement observed following bilateral VIM-DBS (Obwegeser et al., 2000; Ondo et al., 2001; Putzke et al., 2005; Whiting et al., 2018). The present results indicate that bilateral PSA-DBS could provide a valuable alternative treatment for medically intractable ET, warranting further research into its clinical utility and comparative effectiveness.

Moreover, bilateral PSA-DBS was associated with mild and tolerable side effects in this study. Although dysarthria (*n* = 4) and balance disorder (*n* = 2) emerged in the initial stages of treatment, all adverse side effects were transient and subsequently resolved or reduced by DBS reprograming without affecting the therapeutic effects (Table 5). Indeed, previous studies have similarly shown that decreasing the pulse width or frequency or other parameter adjustments offer a powerful means to eliminate or reduce adverse side effects while maintaining the clinical benefits of DBS treatment to patients (Ramirez-Zamora et al., 2016; Choe et al., 2018; Moldovan et al., 2018). Thus, bilateral PSA-DBS could provide not only an effective but also a relatively safe treatment for refractory ET.

**TABLE 5:** Stimulation parameters at 6-month and last follow-up^#^.

**Patient**		**Six months**				**Last follow-up**		
		**Voltage (V)**	**Frequency (Hz)**	**Pulse width (μs)**		**Voltage (V)**	**Frequency (Hz)**	**Pulse width (μs)**
Patient 1	C + 4−5−	2.25	125	60	C + 4−	1.75	125	60
	C + 0−1−	2.25	115	60	C + 0−	2.45	115	60
Patient 2	C + 8−9−	2.5	160	60	C + 8−9−	2.5	160	60
	C + 0−1−	2.5	160	60	C + 0−1−	2.5	160	60
Patient 3	C + 8−	2.9	160	70	C + 8−9−	3.0	135	60
	C + 1−	2.3	160	60	C + 1−	2.35	135	60
Patient 4	C + 6−	2.15	135	50	C + 6−	2.15	135	50
	C + 2−	2.1	135	50	C + 2−	2.0	135	50
Patient 5	C + 6−5−	3.35	135	60	C + 6−5−	3.35	135	60
	C + 1−0−	2.15	135	60	C + 1−0−	2.15	135	60
Patient 6	C + 6−5−	2.25	145	80	C + 6−5−	2.65	145	80
	C + 1−	1.75	145	60	C + 1−	2.35	145	60
Patient 7	C + 5−6−	2.15	145	60	C + 5−6−	2.35	145	60
	1−2 +	2.65	145	80	1−2 +	2.65	145	80

*^#^Six patients were followed up to 12 months; 1 patient was followed up to 9 months.*

It was concerning, however, that one of the patients developed paralysis in the right arm due to edema in the trajectory of the left DBS lead. This adverse event was linked to the close proximity between the electrode trajectory and motor cortex. To adjust this, we used a large coronal and sagittal angle so that the electrodes crossed posterior to the central gyrus and encompassed both the PSA and VIM in one trajectory. Subsequently, the patient’s paralysis was resolved at 6-month follow-up.

The results of this case series are promising but should be considered as tentative and preliminary given the study limitations. The study included only a small patient sample, did not include a comparison group, and had significant potential for bias and confounding. Accordingly, the results may be categorized as class IV evidence. In addition, the patient sample was clinically heterogenous and the DBS device used was not identical in all patients. Thus, additional research is required to support or refute the present findings before any firm conclusions can be drawn.

## Conclusion

This study indicated that bilateral PSA-DBS could offer an effective and safe alternative treatment for at least some cases of medically intractable ET. The results seem to us sufficiently promising to warrant the initiation of larger and well-controlled studies to assess its clinical utility, adverse-event profile, and comparative effectiveness.

## Data Availability Statement

All datasets generated for this study are included in the article/supplementary material.

## Ethics Statement

Written informed consent was obtained from the individual(s) for the publication of any potentially identifiable images or data included in this article.

## Author Contributions

DL, YW, BS, and CZ performed the concept and design. XS and LH performed the data acquisition. XS, LH, and YW performed the analysis and interpretation of data. DL and YW performed the statistical review and critique. XS, LH, CZ, DL, YW, YP, TW, JD, and HL drafted the article. XS, DL, and YW approved the final submitted version.

## Conflict of Interest

The authors declare that the research was conducted in the absence of any commercial or financial relationships that could be construed as a potential conflict of interest.

## References

[B1] Anderson D. Kartha N. (2013). Deep brain stimulation in nonparkinsonian movement disorders and emerging technologies, targets, and therapeutic promises in deep brain stimulation. *Neurol. Clin.* 31 809–826. 10.1016/j.ncl.2013.03.008 23896507

[B2] Barbe M. Liebhart L. Runge M. Deyng J. Florin E. Wojecki L. (2011). Deep brain stimulation of the ventral intermediate nucleus in patients with essential tremor: stimulation below intercommissural line is more efficient but equally effective as stimulation above. *Exp. Neurol.* 230 131–137. 10.1016/j.expneurol.2011.04.005 21515262

[B3] Barbe M. T. Reker P. Hamacher S. Franklin J. Kraus D. Dembek T. A. (2018). DBS of the PSA and the VIM in essential tremor: a randomized, double-blind, crossover trial. *Neurology* 91 543–550. 29970404 10.1212/WNL.0000000000005956

[B4] Benabid A. L. Benazzouz A. Hoffmann D. Limousin P. Krack P. Pollak P. (1998). Long-term electrical inhibition of deep brain targets in movement disorders. *Mov. Disord.* 13 119–125. 10.1002/mds.870131321 9827607

[B5] Benabid A. L. Pollak P. Gao D. Hoffmann D. Limousin P. Gay E. (1996). Chronic electrical stimulation of the ventralis intermedius nucleus of the thalamus as a treatment of movement disorders. *J. Neurosurg.* 84 203–214. 10.3171/jns.1996.84.2.0203 8592222

[B6] Bhatia K. P. Bain P. Bajaj N. Elble R. J. Hallett M. Louis E. D. (2018). Consensus statement on the classification of tremors, from the task force on tremor of the international parkinson and movement disorder society. *Mov. Disord.* 33 75–87. 10.1002/mds.27121 29193359 PMC6530552

[B7] Blomstedt P. Sandvik U. Hariz M. I. Fytagoridis A. Forsgren L. Hariz G. M. (2011). Influence of age, gender and severity of tremor on outcome after thalamic and subthalamic DBS for essential tremor. *Parkinsonism Relat. Disord.* 17 617–620. 10.1016/j.parkreldis.2011.05.014 21676643

[B8] Blomstedt P. Sandvik U. Tisch S. (2010). Deep brain stimulation in the posterior subthalamic area in the treatment of essential tremor. *Mov. Disord.* 25 1350–1356. 10.1002/mds.22758 20544817

[B9] Bot M. van Rootselaar F. Contarino M. F. Odekerken V. Dijk J. (2017). Deep brain stimulation for essential tremor: aligning thalamic and posterior subthalamic targets in 1 surgical trajectory. *Oper. Neurosurg.* 15 144–152. 10.1093/ons/opx232 29281074

[B10] Choe C.-U. Hiding U. Schaper M. Gulberti A. Köppen J. Buhmann C. (2018). Thalamic short pulse stimulation diminishes adverse effects in essential tremor patients. *Neurology* 91 704–713. 10.1212/WNL.0000000000006033 30045955

[B11] Fahn S. Tolosa E. Marín C. (1988). “Clinical rating scale for tremor,” in *Parkinson’s Disease and Movement Disorders*, eds Jankovic J. Tolosa E., (Munich: Urban & Schwarzenberg), 225–234.

[B12] Flora E. D. Perera C. L. Cameron A. L. Maddern G. J. C. (2010). Deep brain stimulation for essential tremor: a systematic review. *Mov. Disord.* 25 1550–1559. 10.1002/mds.23195 20623768

[B13] Fytagoridis A. Blomstedt P. (2010). Complications and side effects of deep brain stimulation in the posterior subthalamic area. *Stereotact. Funct. Neurosurg.* 88 88–93. 10.1159/000271824 20068384

[B14] Fytagoridis A. Sandvik U. Aström M. Bergenheim T. Blomstedt P. (2012). Long term follow-up of deep brain stimulation of the caudal zona incerta for essential tremor. *J. Neurol. Neurosurg. Psychiatry.* 83 258–262. 10.1136/jnnp-2011-300765 22205676

[B15] Ghilardi M. G. D. S. Ibarra M. Alho E. J. L. Reis P. R. Lopez Contreras W. O. Hamani C. (2018). Double target DBS for essential tremor: 8-contact lead for CZI and Vim aligned in the same trajectory. *Neurology* 90 476–478. 10.1212/wnl.0000000000005076 29438045

[B16] Herzog J. Hamel W. Wenzelburger R. Monika P. Marcus O. P. Bartussek J. (2007). Kinematic analysis of thalamic versus subthalamic neurostimulation in postural and intention tremor. *Brain* 130(Pt 6) 1608–1625. 10.1093/brain/awm077 17439979

[B17] Kitagawa M. Murata J. Kikuchi S. Sawamura Y. Saito H. Sasaki H. (2000). Deep brain stimulation of subthalamic area for severe proximal tremor. *Neurology* 55 114–116. 10.1212/wnl.55.1.114 10891917

[B18] Louis E. D. Ferreira J. J. (2010). How common is the most common adult movement disorder? Update on the worldwide prevalence of essential tremor. *Mov. Disord.* 25 534–541. 10.1002/mds.22838 20175185

[B19] Mitchell K. T. Paul L. Starr P. A. Okun M. S. Wharen R. E. (2000). Benefits and risks of unilateral and bilateral ventral intermediate nucleus deep brain stimulation for axial essential tremor symptoms. *Parkinsonism Relat. Dis.* 60 126–132. 10.1016/j.parkreldis.2018.09.004 30220556

[B20] Moldovan A.-S. Hartmann C. J. Trenado C. Meumertzheim N. Slotty P. J. Vesper J. (2018). Less is more – pulse width dependent therapeutic window in deep brain stimulation for essential tremor. *Brain Stimul.* 11 1132–1139. 10.1016/j.brs.2018.04.019 29735344

[B21] Obwegeser A. A. Uitti R. J. Turk M. F. Strongosky A. J. Wharen R. E. (2000). Thalamic stimulation for the treatment of midline tremors in essential tremor patients. *Neurology* 54 2342–2344. 10.1212/wnl.54.12.2342 10881269

[B22] Ondo W. Almaguer M. Jankovic J. Simpson R. K. (2001). Thalamic deep brain stimulation: comparison between unilateral and bilateral placement. *Arch. Neurol.* 58 218–222. 11176959 10.1001/archneur.58.2.218

[B23] Pahwa R. Lyons K. E. Wilkinson S. B. Simpson R. K. Jr. Ondo W. G. Tarsy D. (2006). Long-term evaluation of deep brain stimulation of the thalamus. *J. Neurosurg.* 104 506–512. 16619653 10.3171/jns.2006.104.4.506

[B24] Papavassiliou E. Rau G. Heath S. Abosch A. Barbaro N. M. Larson P. S. (2004). Thalamic deep brain stimulation for essential tremor: relation of lead location to outcome. *Neurosurgery* 54 1120–1129; discussion 1129–1130. 15113466 10.1227/01.neu.0000119329.66931.9e

[B25] Patric B. Ulrika S. Anders F. Stephen T. (2009). The posterior subthalamic area in the treatment of movement disorders: past, present, and future. *Neurosurgery* 64 1029–1042. 10.1227/01.NEU.0000345643.69486.BC 19487881

[B26] Pilitsis J. G. Metman L. V. Toleikis J. R. Hughes L. E. Sani S. B. Bakay R. A. (2008). Factors involved in long-term efficacy of deep brain stimulation of the thalamus for essential tremor. *J. Neurosurg.* 109 640–646. 10.3171/JNS/2008/109/10/0640 18826350

[B27] Putzke D. J. Uitti R. J. Obwegeser A. A. Wszolek Z. K. Wharen R. E. (2005). Bilateral thalamic deep brain stimulation: midline tremor control. *J. Neurol. Neurosurg. Psychiatry* 76 684–690. 10.1136/jnnp.2004.041434 15834027 PMC1739619

[B28] Ramirez-Zamora A. Boggs H. Pilitsis J. G. (2016). Reduction in DBS frequency improves balance difficulties after thalamic DBS for essential tremor. *J. Neurol. Sci.* 367 122–127. 10.1016/j.jns.2016.06.001 27423573

[B29] Shih L. C. Kathrin L. F. Chen L. Efstathios P. Tarsy D. (2013). Loss of benefit in VIM thalamic deep brain stimulation (DBS) for essential tremor (ET): how prevalent is it? *Parkinsonism Relat. Dis.* 19 676–679. 10.1016/j.parkreldis.2013.03.006 23582712

[B30] Ulrika S. Lars-Owe K. Anders L. Patric B. (2012). Thalamic and subthalamic deep brain stimulation for essential tremor: where is the optimal target? *Neurosurgery* 70 840–846. 10.1227/neu.0b013e318236a809 22426044

[B31] Whiting B. B. Whiting A. C. Whiting D. M. (2018). Thalamic deep brain stimulation. *Prog. Neurol. Surg.* 33 198–206. 10.1159/000481104 29332084

[B32] Xie T. Bernard J. Warnke P. (2012). Post subthalamic area deep brain stimulation for tremors: a mini-review. *Transl. Neurodegener.* 1:20. 10.1186/2047-9158-1-20 23210767 PMC3534556

